# What Task Feature Determines the Dominant Task in Dual-Task Conditions?

**DOI:** 10.1523/ENEURO.0542-24.2025

**Published:** 2025-03-19

**Authors:** Lu Gan, Zhilin Zhang, Zhiting Zhang, Jinglong Wu, Ji Dai, Shintaro Funahashi

**Affiliations:** ^1^Research Center for Medical Artificial Intelligence, Shenzhen Institute of Advanced Technology, Chinese Academy of Sciences, Shenzhen 518055, China; ^2^Shenzhen Technological Research Center for Primate Translational Medicine, Shenzhen-Hong Kong Institute of Brain Science, Shenzhen Institute of Advanced Technology, Chinese Academy of Sciences, Shenzhen 518055, China; ^3^CAS Key Laboratory of Brain Connectome and Manipulation, the Brain Cognition and Brain Disease Institute, Shenzhen Institute of Advanced Technology, Chinese Academy of Sciences, Shenzhen 518055, China; ^4^Guangdong Provincial Key Laboratory of Brain Connectome and Behavior, Shenzhen Institute of Advanced Technology, Chinese Academy of Sciences, Shenzhen 518055, China; ^5^University of Chinese Academy of Sciences, Beijing 100049, China

**Keywords:** dual-task interference, memory management, monkey, task-order effect, working memory

## Abstract

When attempting to concurrently perform two distinct cognitive tasks, the performance of either task is frequently compromised. This phenomenon is known as dual-task interference. Although multiple task features have been postulated to influence on dual-task interference, the primary determinant remains unclear. The determinant factor causing dual-task interference is an important issue to understand its mechanism and associated functions including switching tasks and planning task order. The present study investigated this issue using monkeys and three behavioral tasks requiring distinct cognitive processes (spatial working memory, SWM; working memory and long-term memory of objects, PA; object working memory, DMS) and manipulating task pair (SWM and PA or SWM and DMS), task order (fixed or randomized), and task difficulty (different delay lengths). The task introduced first showed better performance as compared with the task introduced second, suggesting the task order as an important factor. However, the performance of the SWM task decreased when preceded by the PA and DMS tasks, while the latter tasks were unaffected when the SWM task was introduced first. This tendency was more obvious in random-order conditions than fixed-order conditions. Further, interference effect increased as task difficulty increased. Although the task order is one determinant, our results show the difference in cognitive process needed for tasks, its complexity, and the demand of working memory resources as more significant determinants for deciding the dominant task in dual-task conditions, indicating importance of neural mechanisms including managing working memory resources and coordinating multiple cognitive processes to understand the cause of dual-task interference.

## Significance Statement

Although multiple task features have been postulated to influence on dual-task interference, the primary cause of dual-task interference remains unclear. Using monkeys and three behavioral tasks, each requiring distinct cognitive processes, and manipulating task pairs, task order, and task difficulty, we found the difference in necessary cognitive processes among tasks, complexity and difficulty of tasks, and fixed- or random-order conditions as critical task factors causing dual-task interference. These task factors are closely related to the mechanisms how different cognitive processes are coordinated and how finite working memory resources are managed and are associated with executive control. Dual-task paradigms are the best method to examine neural mechanism of executive control.

## Introduction

When we perform two cognitive tasks simultaneously (dual-task condition), one of the component tasks demonstrates reduced performance while the other task maintains normal or near-normal performance, despite our efforts to perform each task accurately. This phenomenon is referred to as dual-task interference (Kahneman, 1973; Navon and Miller, 1987; Pashler, 1994; Wickens, 2002). Dual-task interference has been interested in cognitive neuroscience, because this effect is closely related to effective management of the limited capacity of cognitive resources for information processing (Kahneman, 1973; Duncan, 1980; Wickens, 2002) and because the cause of this effect is related to executive control, such as switching or dividing attention, task coordination, and planning (Miyake and Shah, 1999; Funahashi, 2022a).

Although several models have been proposed (Meyer and Kieras, 1997), two models have been known to explain the phenomenon of dual-task interference: the capacity sharing model (Kahneman, 1973; Wickens, 2002) and the bottleneck model (Pashler, 1994). The capacity sharing model attributes dual-task interference to the finite capacity of cognitive resources for performing various cognitive activities (Kahneman, 1973; Duncan, 1980; Wickens, 2002). When two different cognitive tasks are carried out simultaneously, interference occurs if both tasks compete for the same limited cognitive resources and surpass the total available resource. Since the amount of working memory resources is limited (Cowan, 2001; Marois and Ivanoff, 2005; Baddeley, 2012), which task feature determines the amount of working memory resources needed for each task is a crucial issue to understand dual-task interference. When verbal and visual tasks were involved in dual-task experiments, a stronger interference effect was observed when both memory sets belonged to the same information domain (e.g., visual) compared with when they belonged to different domains (e.g., visual vs verbal; Cowan and Morey, 2007). Further, the auditory task was found to be worse in dual-task conditions in which simultaneous rapid visual and auditory stimulus sequences were used (Kasper et al., 2014). These emphasize that processing different modality of information requires different amounts as well as different types of cognitive resources. However, if the auditory task imposes higher cognitive demands than the visual task, the auditory task becomes dominant in dual-task conditions (Gherri and Eimer, 2011), indicating that the priority of the task and task difficulty are also important factors to allocate memory resources. On the other hand, the strength of dual-task interference is greater when two tasks share many common features as opposed to few features (Cocchini et al., 2002; Fougnie and Marois, 2006). When two tasks require the common mechanism simultaneously, a decrease in the performance of one or both tasks would be observed due to the bottleneck effect (bottleneck model; Pashler, 1994). In the bottleneck model, the processing order is determined by the arrival timings of stimuli at the bottleneck, therefore following a first-come-first-serve principle (De Jong, 1995; Stroback et al., 2018). The evidence supporting the bottleneck model has been observed repeatedly by psychological refractory period (PRP) paradigms (Jiang, 2004; Hirsch et al., 2018). However, comparing behavioral performances between fixed- and random-order conditions showed that behavioral performances deteriorated in random-order conditions (Kubler et al., 2022), suggesting that the task order effect is not solely attributed to the bottleneck mechanism but also to working memory mechanisms for maintaining task-order information and flexible scheduling by executive processes (Meyer and Kieras, 1997).

Thus, although multiple task features have been postulated to influence on dual-task interference, the primary determinant of dual-task interference remains unclear. Understanding what task feature is critical for a task to become the more successful (“the winner”) in the dual-task condition is a crucial issue in comprehending the mechanism of dual-task interference (Klingberg, 2000; Gan et al., 2022; Funahashi, 2022b) as well as executive control (Baddeley, 1986, 2012; Funahashi, 2022b). In the present study, to discover the critical task factor to determine the winner in dual-task conditions by systematic behavioral experiments, we used rhesus monkeys as subjects and three cognitive tasks (spatial and object working memory tasks and object paired association task), each requiring the maintenance of distinct information in working memory and diverse information processing for execution. We selected two among three tasks for dual-task experiments, presented them in either fixed- or random-order, and manipulated task difficulty by varying the delay lengths for each task to identify the determining factor for the dominant task in dual-task conditions.

## Materials and Methods

### Subjects

Three male rhesus monkeys (A, 7.5 kg; B, 8.8 kg; C, 9.0 kg) were used for this study. Each monkey was housed in individual cage in a primate facility, where food was freely accessible. While water intake was restricted in their home cages, they could obtain daily required amount of water as reward in the laboratory. If necessary, additional water, fruit, or vegetables were provided in their cages after the experiment. All research procedures were conducted in accordance with the regulations set in the Guide for the Care and Use of Laboratory Animals (Eighth Edition, 2011) and were approved by the Institutional Animal Care and Use Committee at the Shenzhen Institutes of Advanced Technology, Chinese Academy of Sciences.

### Experimental apparatus

During the experiment, monkeys were positioned in primate chairs in a dimly lit, sound-attenuated room with their head movements restricted by a head-restraining instrument attached on the skull. Visual stimuli were displayed on a 24 inch LED monitor (ASUS VG248), placed 57 cm in front of the monkey's face. Eye movements were sampled using an eye tracker (jsmzEM2000C) at 1,000 Hz. A 13-point calibration was carried out at the beginning and midpoint of the experiment to ensure accurate eye tracking. To control the monkey's behavior, present visual stimuli, deliver the reward, and collect behavioral data during the experiment, the program named MonkeyLogic, a MATLAB-based toolbox developed by the National Institute of Mental Health (Bethesda, USA), was employed.

### Single-task conditions of SWM and PA tasks

**Figure 1.**
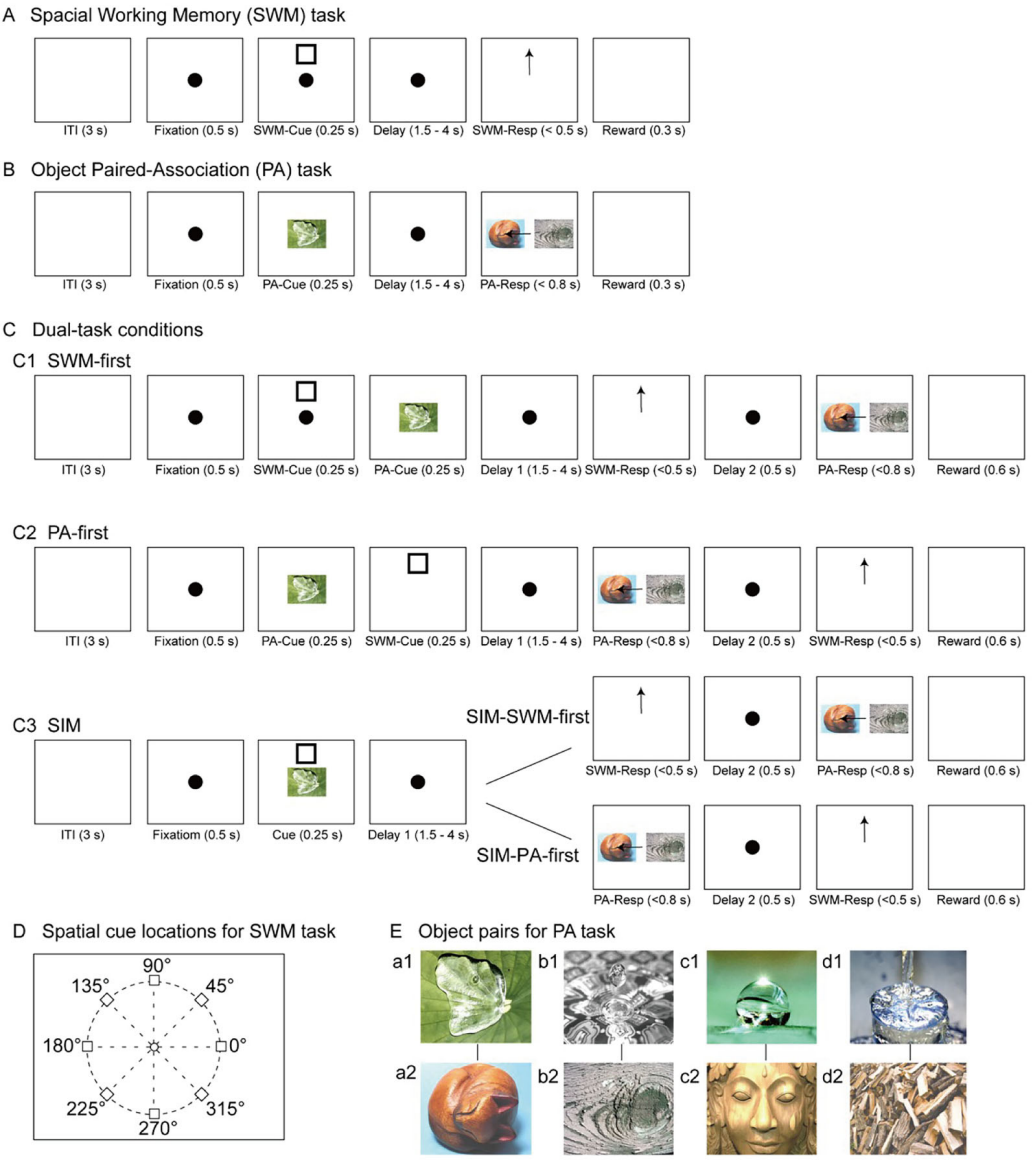
Illustration of the experimental paradigms used the SWM task and the PA task as component tasks. ***A***, The spatial working memory (SWM) task in a single-task condition. The subject was required to retain the position of a spatial cue during a delay period. ***B***, The object pair association (PA) task in a single-task condition. The subject was required to keep four pairs of objects in long-term memory and retrieve the paired associate of the object cue after a delay period. ***C***, The dual-task conditions using both SWM and PA tasks under fixed-order conditions (SWM-first and PA-first) and random-order conditions (SIM-SWM-first and SIM-PA-first). In the fixed-order conditions, the SWM-cue and PA-cue were sequentially presented in a fixed order, and the subject needed to execute eye movement responses in the same order as the cue presentation. In the random-order conditions (SIM), both SWM-cue and PA-cue were presented simultaneously, and the subject needed to perform either the SWM task or PA task first based on the absence or presence of corresponding visual stimuli on the monitor after the delay period. ***D***, The positions of the spatial cue for the SWM task. ***E***, The four sets of object pairs for the PA task. Arrows in the figures indicate the directions of the correct eye movements. See Extended Data Figure 1-1 for more details.

The primary tasks for the experiment were a spatial working memory (SWM) task and an object paired association (PA) task. In the single-task condition of the SWM task (Fig. 1*A*), a 3 s intertrial interval (ITI) was followed by the presentation of a fixation point (a white circle, 0.2° in visual angle) at the center of the monitor. The monkey's task was to maintain fixation on the fixation point. After maintaining fixation for 0.5 s (fixation period), a spatial cue (a white square, 1° in visual angle) appeared for 0.25 s (cue period) at one of eight predetermined peripheral positions (Fig. 1*D*). The position of the spatial cue was randomly selected for each trial. Subsequently, the delay period started and the monkey had to continue fixating on the fixation point. The duration of the delay period ranged randomly from 1.5 to 4 s. When the delay period ended, the fixation point disappeared, signaling the monkey to make a saccade to the cued location within 0.5 s (response period). If the monkey executed a correct saccade and maintained its gaze within a circular window surrounding the spatial cue (target window, 3° in diameter) for 0.5 s (hold period), it would receive a drop of juice as a reward.

10.1523/ENEURO.0542-24.2025.f1-1 Figure 1-1 Illustration of the experimental paradigms used the SWM task and the DMS task as component tasks. (A) Spatial working memory (SWM) task in single-task condition. This task requires to maintain a spatial cue position during the delay period. (B) Object delayed matching-to-sample (DMS) task in single-task condition. This task requires to maintain the object cue after the delay period. (C) Dual-task conditions using SWM and DMS tasks. Dual-task conditions include two fixed-order conditions (SWM-first and DMS-first) and two random-order (SIM) conditions (SIM-SWM-first and SIM-DMS-first). In fixed-order conditions, SWM-cue and DMS-cue were presented sequentially in fixed order either in SWM-cue first or DMS-cue first, and the subject performed eye movement responses with the same order as the cue presentation. In random-order conditions, both SWM-cue and DMS-cue were presented simultaneously, and subjects performed either SWM task or DMS task first depending on whether no visual stimulus was presented (SIM-SWM-first) or two object stimuli were presented (SIM-DMS-first) on the monitor after the delay period. (D) Positions of the spatial cue for the SWM task. (E) Eight objects for the DMS task. Arrows in figures indicate directions of correct eye movements. Download Figure 1-1, TIF file.

In the PA task (Fig. 1*B*), a set of eight images was chosen, comprising four pictures of wooden objects and four depicting water scenes. These images were then paired to form four distinct pairs, with each pair consisting of one wooden object and one water scene (Fig. 1*E*). During the PA task, monkeys were tasked to identify the correct paired picture corresponding to a cued picture within the 0.8 s response period. Success required monkeys to memorize and recall these specific picture associations to receive a reward. Following a 3 s ITI, a fixation point appeared at the center of the monitor. After maintaining fixation on this point for 0.5 s (fixation period), one cued picture (1° in visual angle) randomly selected from the pool of eight images was displayed for 0.25 s (cue period) over the fixation point. Subsequently, a delay period lasting between 1.5 and 4 s was introduced and monkeys had to maintain fixation on the fixation point. At the end of the delay period, two images were presented, one being the picture paired with the cued picture (target picture) while the other served as a distractor selected from the remaining 6 pictures. The positions of the target picture and the distractor were randomized across trials, and monkeys were required to shift their gaze to the target picture within 0.8 s (response period). Successfully maintaining fixation on the target picture for 0.5 s (hold period) enabled monkeys to receive a reward. SWM and PA tasks were separately trained in single-task conditions. The dual-task training using these two tasks began after achieving a performance criterion of over 80% accuracy in three consecutive training sessions for both the SWM and PA tasks under single-task conditions.

### Dual-task conditions of SWM and PA tasks

In dual-task conditions, SWM and PA tasks were administered in three different sequences: SWM-first, PA-first, and simultaneous (SIM; Fig. 1*C*). In the SWM-first condition (Fig. 1*C1*), following a 3 s ITI, monkeys were first needed to fixate on a fixation point for 0.5 s (first fixation period). Subsequently, while monkeys were maintained fixation, a spatial cue for the SWM task appeared for 0.25 s at one of eight positions, followed by the presentation of a cue picture for the PA task chosen from eight pictures for another 0.25 s (0.5 s cue period). During the delay period (1.5–4 s), monkeys had to maintain fixation on the fixation point. At the end of the delay period, the fixation point vanished, serving as the Go signal prompting monkeys to execute a saccade to the cued position within 0.5 s (response period of the SWM task). Subsequently, irrespective of the accuracy of the saccade, the fixation point reappeared at the center of the monitor, and monkeys were required to maintain fixation for another 0.5 s (second fixation period). At the end of the second fixation period, two pictures (a target picture and a distractor) were simultaneously displayed on the left and right positions (response period of the PA task). Monkey were required to select the target picture within 0.8 s. Only when monkeys made correct responses in both tasks, they received a reward. Correct response only on the PA task would not provide monkeys the reward.

In the PA-first condition (Fig. 1*C2*), the sequence and timings of task events were the same as those of the SWM-first condition. However, a cue picture for the PA task was displayed for 0.25 s, followed by a spatial cue for the SWM task that was displayed 0.25 s during the 0.5 s cue period. In the response period, two pictures (a target picture and a distractor) were presented first, prompting monkeys to select the correct target picture by eye movements within 0.8 s (response period of the PA task). Following a 0.5 s fixation period, the fixation point disappeared, signaling monkeys to make a saccade to the correct cued position within 0.5 s (response period of the SWM task). Similar as the SWM-first condition, monkeys only received a reward after providing correct responses in both tasks and would not receive a reward even if monkeys made a correct saccade in the SWM task.

In the SIM condition (Fig. 1*C3*), the order and timings of task events closely resembled those of the SWM-first and PA-first conditions. However, in the SIM condition, a cue picture for the PA task and a spatial cue for the SWM task were concurrently presented for 0.25 s (cue period). Selection methods of the cue picture and the spatial cue position were the same as those in single-task conditions. The duration of the delay period ranged from 1.5 to 4 s, similar to that in the SWM-first and PA-first conditions. During the response period, in 50% of trials, the same trial events as those in the SWM-first condition were implemented (SIM-SWM-first), while in the remaining 50% of trials, the same trial events as those in the PA-first condition were introduced (SIM-PA-first). Consistent with the SWM-first and PA-first conditions, monkeys only received a reward after providing correct responses in both tasks and would not receive a reward even if monkeys made a correct choice in either task. Monkeys were aware of the required behavioral response sequence in advance in the SWM-first and PA-first conditions, but they were unable to anticipate the required response behavior sequence in the SIM conditions. Three types of dual-task conditions were presented as separate blocks consisting of 100–120 trials each.

Monkey A successfully completed both tasks in single-task conditions and trials across all three dual-task conditions. However, Monkey B could not complete all SIM conditions. As a result, we analyzed the behavioral data from performances in single-task conditions and all dual-task conditions for Monkey A, and the behavioral data from performances in single-task, the SWM-first, and PA-first conditions for Monkey B.

### Single- and dual-task conditions using SWM and DMS tasks

In addition to explore the behavioral performances of both monkeys in single- and dual-task conditions involving SWM and PA tasks, their responses were also analyzed in similar conditions with SWM and the delayed matching-to-sample (DMS) tasks (Extended Data Fig. 1-1). During the DMS task, a set of eight colored objects (Extended Data Fig. 1-1*E*) were used and monkeys were required to identify and select the same object during the response period as had been shown during the cue period. In the single-task condition (Extended Data Fig. 1-1*B*), after a 3 s ITI, a fixation point was displayed at the center of the monitor. After maintaining fixation for 0.5 s, one randomly selected object (1° in visual angle) was displayed for 0.25 s as the cued object. This was followed by a 3 s delay period and monkeys had to maintain fixation. Subsequently, two pictures, one corresponding to the cued object (target) and the other serving as a distractor, were presented on the left and right sides. Monkeys had to promptly make a saccade to the target picture within 0.8 s and maintain fixation for an additional 0.5 s to receive a reward.

In dual-task conditions, three types of dual-task conditions (SWM-first, DMS-first, and SIM) and two variations of SIM conditions (SIM-SWM-first and SIM-DMS-first) were introduced in a block of trials (Extended Data Fig. 1-1). The temporal sequences of trial events and their durations remained consistent across dual-task conditions involving SWM and DMS tasks, as well as those involving SWM and PA tasks.

### Acquisition of behavior data and their analysis

An analysis was conducted to compare the behavioral performances of monkeys in each component task under single-task and dual-task conditions. The study focused on examining percent correct performance rates, reaction times, and accuracies of saccadic eye movements. The data were analyzed using MATLAB software. Percent correct performance rates were calculated for each session to evaluate the monkeys’ performance in each task condition. During behavioral tasks, errors such as break fixation, incorrect saccade directions, and wrong choices were observed in both single-task and dual-task conditions. The study specifically aimed to investigate interference effects seen in dual-task conditions by assessing the monkeys’ ability to deliver a correct response during the response period of each task. Trials in which monkeys broke fixation during the fixation, cue, and delay periods were excluded when calculating percent correct performance rates. This ensured that only trials in which monkeys properly completed the tasks until the response period were considered for analysis.

In dual-task conditions, we calculated correct performance rates of the two tasks individually. The correct performance rate for the SWM task was determined based on the total number of trials and the number of correctly completed SWM trials, regardless of the accuracy of the PA and DMS trial. In the SWM-first and the SIM-SWM-first conditions, the total number of trials analyzed was the total number of trials that lasted until the response period for either the PA task or the DMS task. On the other hand, the correct performance rate for the PA task and the DMS task was calculated based on the total number of trials and the number of correctly completed PA and DMS trials, regardless of the accuracy of the SWM trial. Similarly, in the PA-first, the SIM-PA-first, the DMS-first, and the SIM-DMS-first conditions, the total number of trials analyzed was the total number of trials that lasted until the response period for the SWM task. Thus, the correct performance rate for the PA or DMS task was determined based on the total number of trials and the number of correctly completed PA or DMS trials, respectively, while the correct performance rate for the SWM task was calculated based on the total number of trials and the number of correctly completed SWM trials.

When analyzing error trials, we focused on two specific types of errors in the present study. Type A errors were those that monkeys failed to catch the correct target position within 0.5 s (in the SWM task) or the target picture within 0.8 s (in the PA and DMS tasks). Type B errors were those that monkeys were unable to maintain gazing on the target position or target picture for the hold period of 0.5 s.

In addition, we conducted an analysis of average reaction times for saccade responses in each task under different conditions. Specifically, we compared the reaction times between single-task and dual-task conditions for each respective task. The reaction time was defined as the duration between the disappearance of the fixation point and the initiation of the saccade for the SWM task and between the presentation of two pictures and the saccade initiation for the PA and DMS tasks. The mean reaction times were calculated based on all correct trials, including eight spatial cue conditions for the SWM task and eight picture conditions for both the PA and DMS tasks.

Additionally, to assess the effect of task difficulty in various dual-task conditions on the SWM task, we analyzed the distribution of saccade endpoints and the accuracy of the saccades during the response period in the SWM task. Saccade accuracy was determined by measuring the distance between the correct spatial target position and the observed gaze position during the hold period.

## Results

### SWM and PA tasks in single-task condition

**Figure 2.**
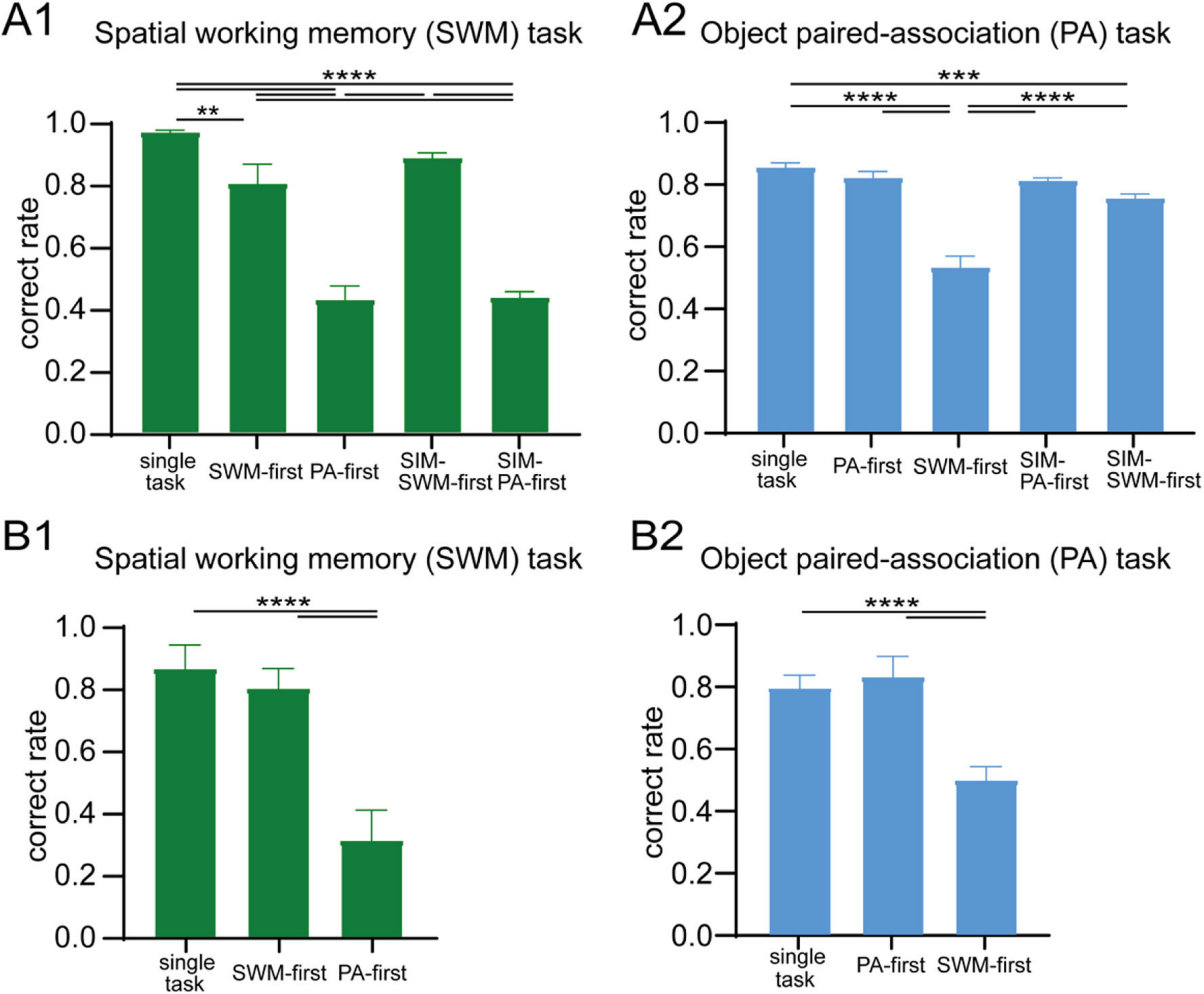
Behavioral results in single-task and dual-task conditions when the SWM task and the PA task were used as component tasks. ***A1***, ***B1***, Correct performance ratio of the SWM task among different task conditions for Monkey A and Monkey B. ***A2***, ***B2***, Correct performance ratio of the PA task among different task conditions for Monkey A and Monkey B. The delay length was 3 s for all task conditions. *p < 0.05, **p < 0.01, ***p < 0.001, ****p < 0.0001. See Extended Data Figure 2-1 for more details.

**Figure 3.**
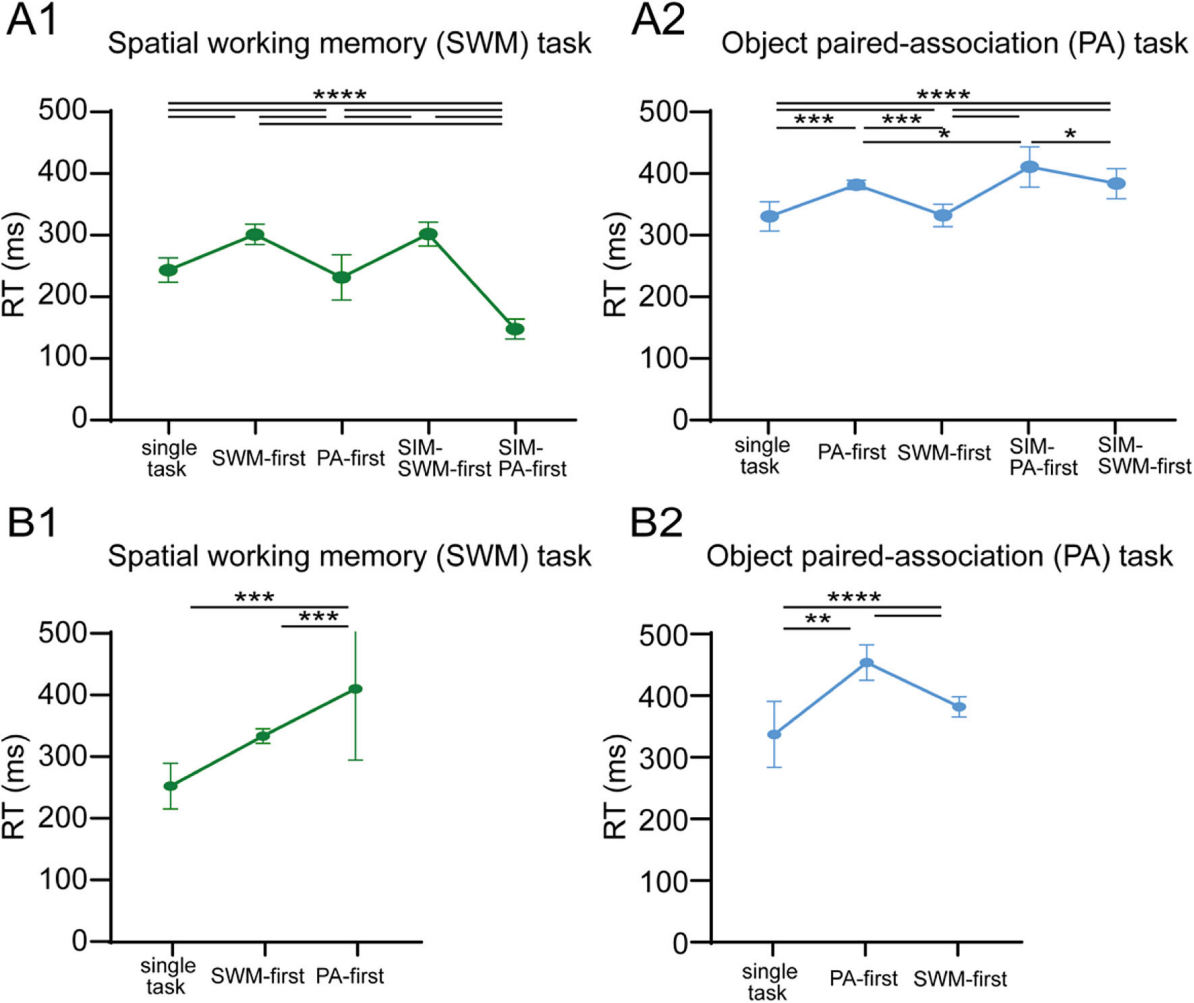
Reaction times in single-task and dual-task conditions when the SWM task and the PA task were used as component tasks. ***A1***, ***B1***, Mean reaction times (RTs) of the SWM task in different task conditions for Monkey A and Monkey B. ***A2***, ***B2***, Mean reaction times of the PA task in different task conditions for Monkey A and Monkey B. The delay length was 3 s for all task conditions. *p < 0.05, **p < 0.01, ***p < 0.001, ****p < 0.0001.

In the single-task condition, monkeys performed the SWM task and the PA task separately (Fig. 1). The SWM task required monkeys to execute a memory-guided saccade to the location of a previously presented spatial cue (Fig. 1*A*), while the PA task required monkeys to select the picture associated with the cued picture by making an eye movement (Fig. 1*B*). We compared behavioral performances of the SWM and PA tasks under 3 s delay conditions. The average correct performance ratio in the SWM task was 96% for Monkey A (Fig. 2*A1*) and 80% for Monkey B (Fig. 2*B1*). In the PA task, an average correct performance ratio was 85% for Monkey A (Fig. 2*A2*) and 77% for Monkey B (Fig. 2*B2*). These results reveal different task difficulty between the SWM and PA tasks in the single-task condition (Monkey A: *t* = 6.385, *p* < 0.001; Monkey B: *t* = 2.319, *p* < 0.05), indicating that the PA task was more challenging for both monkeys compared with the SWM task. Furthermore, this distinction in task difficulty between the SWM and PA tasks remained consistent throughout the training sessions in correct ratios (Monkey A: *t* = 5.471, *p* < 0.001; Monkey B: *t* = 5.138, *p* < 0.05) and reaction times (Monkey A: *t* = 15.77, *p* < 0.001; Monkey B: *t* = 7.937, *p* < 0.05; Extended Data Fig. 2-1). A difference in task difficulty was also evident in the comparison of mean reaction times during the response period, with the SWM task demonstrating significantly shorter reaction times than the PA task for both monkeys (Monkey A, 239.4 vs 328.8 ms, *t* = 10.41, *p* < 0.001; Monkey B, 249.4 vs 349.7 ms, *t* = 3.783, *p* = 0.002; Fig. 3). The correct performance ratio showed no significant differences across various spatial cue positions (*F*_(3.581,42.97)_ = 2.483, *p* = 0.0638) and different object pairs (*F*_(4.487,53.85)_ = 2.150, *p* = 0.08) in single-task conditions (Fig. 5). Thus, although both monkeys exhibited significantly better behavioral performance in both the SWM and PA tasks under single-task conditions, clear differences in task difficulty were observed, with the SWM task performing easier than the PA task for both monkeys.

10.1523/ENEURO.0542-24.2025.f2-1 Figure 2-1 Temporal changes of behavioral Performances of both SWM and PA tasks along different experimental sessions. (A1 and A2) Correct performance ratios (A1) and reaction times (A2) in the SWM task (green) and the PA task (blue) for monkey A. (B1 and B2) Correct performance ratios (B1) and reaction times (B2) in the SWM task (green) and the PA task (blue) for Monkey B. **** p < 0.0001. Download Figure 2-1, TIF file.

### SWM and PA tasks in dual-task condition

The present study examined the performance of SWM and PA tasks under three different dual-task conditions: SWM-first, PA-first, and SIM conditions (Fig. 1*C*). We compared the performance of each SWM and PA task in both single-task and dual-task conditions. Figure 2 shows the average correct performance of each task under the 3 s delay for two monkeys (Monkeys A and B). In the SWM task, the correct performance ratio was significantly lower in dual-task conditions (80% in SWM-first, 88% in SIM-SWM-first, 43% in PA-first, 43% in SIM-PA-first) compared with the single-task condition (96%) in Monkey A (*F*_(4,60)_ = 79.64, *p* < 0.0001; Fig. 2*A*1), indicating the presence of dual-task interference effect. The reduction in correct performance ratio was more pronounced in PA-first (−54%) and SIM-PA-first (−53%) conditions compared with SWM-first (−17%) and SIM-SWM-first (−8%) conditions (Fig. 2*A1*). Similarly, for the PA task, the correct performance ratio was significantly lower in dual-task conditions (81% in PA-first, 81% in SIM-PA-first, 53% in SWM-first, 75% in SIM-SWM-first) compared with the single-task condition (85%) in Monkey A (*F*_(4,60)_ = 37.97, *p* < 0.0001; Fig. 2*A2*). The reduction in correct performance ratio was greater in SWM-first (−33%) compared with PA-first (−4%), SIM-PA-first (−5%), and SIM-SWM-first (−10%) conditions (Fig. 2*A2*). Similar trends were observed in Monkey B. For the SWM task, the correct performance ratio was significantly lower in PA-first (31%) compared with the single-task condition (86%; *F*_(2,37)_ = 174.4, *p* < 0.0001), with a larger decrease in performance observed in PA-first (−55%) compared with SWM-first (−6%) condition (Fig. 2*B1*). For the PA task, the correct performance ratio was significantly reduced in SWM-first condition (50%) compared with the single-task condition (79%; *F*_(2,42)_ = 204.7, *p* < 0.0001), with a larger decrease in performance observed in SWM-first (−30%) compared with PA-first (+4%) condition (Fig. 2*B2*).

These results reveal that dual-task interference was evident when monkeys engaged in both SWM and PA tasks simultaneously. Under dual-task conditions, the task introduced first consistently performed better than the task introduced second, indicating that the task order plays a significant role in determining task performance. However, when the correct performance ratio was compared between SWM and PA tasks when these tasks were performed second, the reduction of the SWM task was more pronounced in the PA-first condition (−54% for Monkey A and −55% for Monkey B) and the SIM-PA-first condition (−53% for Monkey A) compared with the PA task in the SWM-first condition (−17% for Monkey A and −30% for Monkey B) and the SIM-SWM-first condition (−8% for Monkey A), showing a greater impact of interference effect on the SWM task than on the PA task for both monkeys when behavioral responses of the SWM task were performed second. Behavioral results in single-task conditions showed that the SWM task is easier than the PA task. In addition, interference effect was different between fixed-order and random-order conditions. These results indicate that, although task order plays an important role, differences of information processing necessary for each task, task difficulty, and either fixed-order or random-order conditions play more significant roles to determine the dominant task in dual-task conditions.

### Comparison of reaction times between SWM and PA tasks

To investigate the impact of interference on saccadic eye movements, we analyzed reaction times during the response period for each task (Fig. 3). In the SWM task, significant difference in reaction times was observed between the SWM-first condition (297.5 ms) and the SIM-SWM-first condition (297.9 ms) compared with the single-task condition (239.4 ms) for Monkey A (Fig. 3*A1*) and Monkey B (330.6 ms in WM-first and 249.4 ms in single-task condition; Fig. 3*B1*). Similarly, in the PA task, significant difference was observed between the PA-first condition (380.1 ms) and the SIM-PA-first condition (409.3 ms) compared with the single-task condition (328.8 ms) for Monkey A (Fig. 3*A2*) and Monkey B (378.8 ms in PA-first and 349.7 ms in single-task condition; Fig. 3*B2*). Although significant task order effect was observed on motor responses, there were significant contrarily changes in reaction times compared with those in the single-task condition when the task was performed second. In Monkey A, the introduction of the SWM task as the second task led to significantly shorter reaction times (227.7 ms in PA-first and 143.9 ms in SIM-PA-first conditions) compared with the single-task condition (239.4 ms; *F*_(2,36)_ = 72.71, *p* < 0.0001; Fig. 3*A1*). However, when the PA task was introduced secondly, reaction times for the PA task became significantly longer in the SIM-SWM-first condition (382.2 ms) compared with the single-task condition (328.8 ms; *F*_(2,36)_ = 25.32, *p* < 0.0001; Fig. 3*A2*). Conversely, in Monkey B, reaction times for both SWM and PA tasks became significantly longer in the PA-first condition (330.6 vs 249.4 ms; *t* = 8.871, *p* < 0.0001; Fig. 3*B1*) and in the WM-first condition (378.8 vs 349.7 ms; *F*_(2,42)_ = 32.86, *p* = 0.0502; Fig. 3*B2*). These results indicate that dual-task interference can impact on motor responses when the task is performed either first or second. However, the direction (increase or decrease) and the magnitude of interference effect on motor responses vary depending on the subjects and the task order and the task difference.

### Effect of delay length on dual-task interference

**Figure 4.**
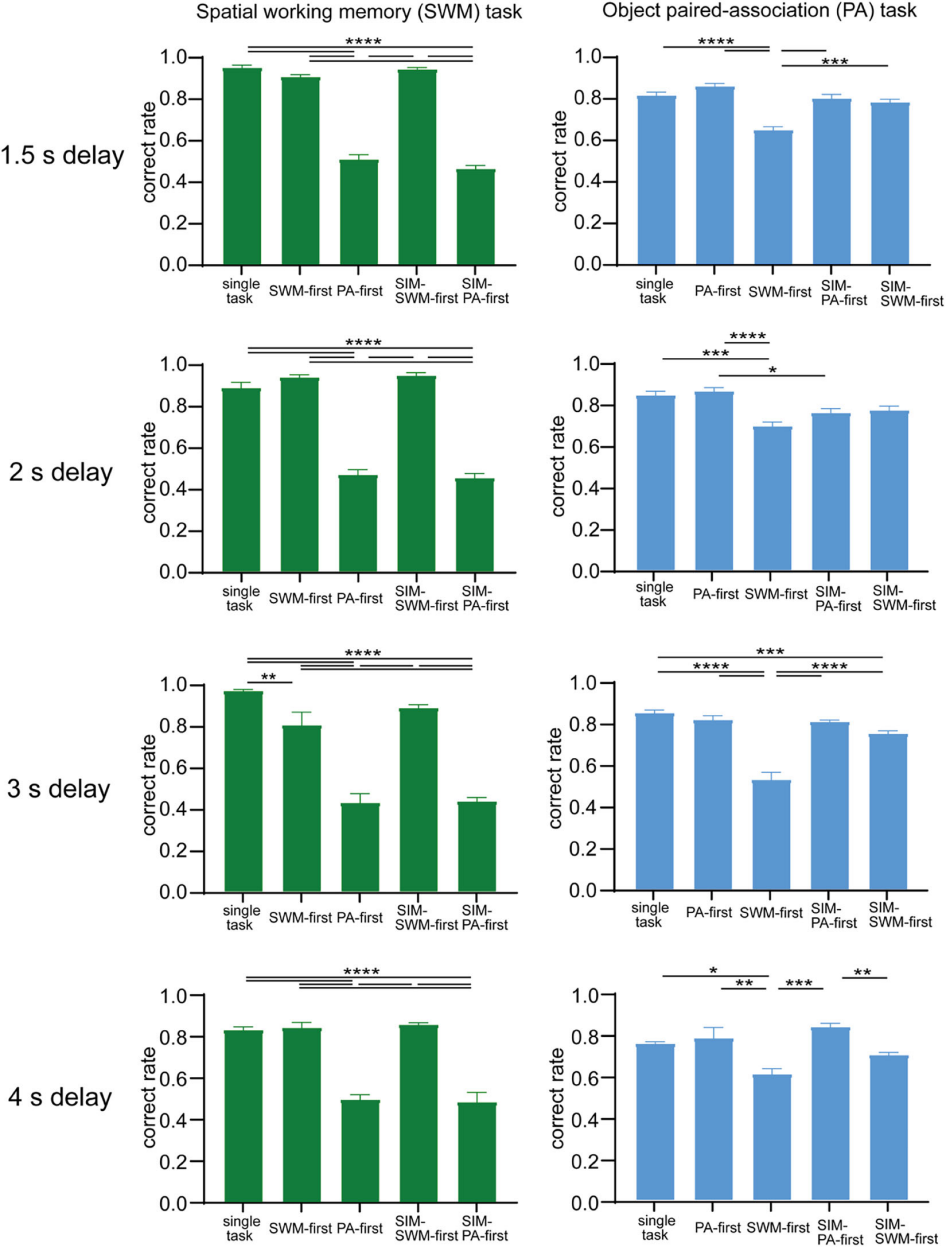
Effects of task difficulty on behavioral performances of the SWM task and the PA task under different task conditions in Monkey A. Task difficulty was controlled by changing the delay length between 1 and 4 s. Behavioral data for each delay length were obtained in separate blocks of trials. See Extended Data Figure 4-1 for more details.

**Figure 5.**
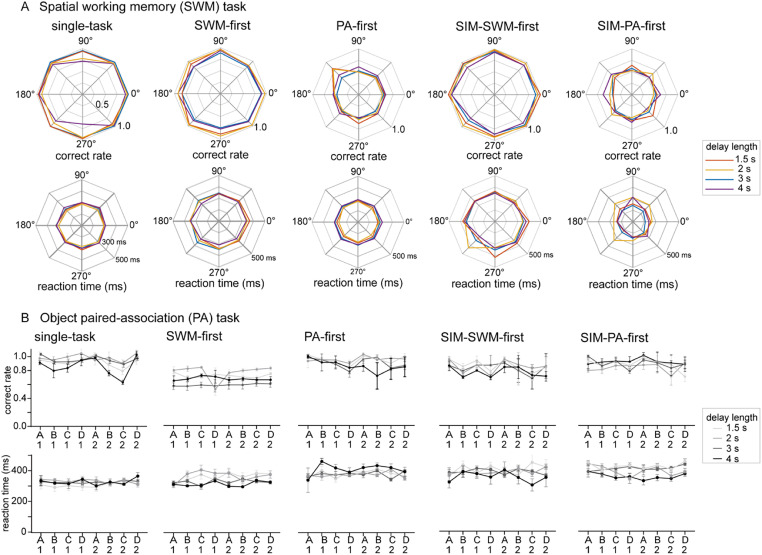
Behavioral performances of the SWM and the PA task under different spatial and object conditions. ***A***, Correct performance ratios (top figures) and reactions times (bottom figures) across eight different spatial cue positions in the SWM task. Different colors indicate data obtained in different delay lengths. No significant differences were observed in correct performance ratios except 4 s delay (1.5 s, *F*_(3.121,15.60)_ = 1.045, *p* = 0.4027; 2 s, *F*_(2.263,9.053)_ = 2.328, *p* = 0.15; 3 s, *F*_(3.581,42.97)_ = 2.483, *p* = 0.0638; 4 s, *F*_(2.079,12.47)_ = 4.08, *p* < 0.05) and in reaction times (1.5 s, *F*_(1.432,7.160)_ = 2.813, *p* = 0.1321; 2 s, *F*_(2.275,9.100)_ = 1.377, *p* = 0.3041) except 3 s (*F*_(3.044,36.53)_ = 8.881, *p* < 0.0001) and 4 s (*F*_(2.203,13.22)_ = 4.654, *p* < 0.05) across different spatial conditions. ***B***, Correct performance ratios (top figures) and reactions times (bottom figures) across eight different object stimuli in the PA task. Different line shapes indicate data obtained in different delay lengths. No significant differences were observed in correct performance ratios (1.5 s, *F*_(3.173,15.86)_ = 1.424, *p* = 0.2727; 2 s, *F*_(2.398,9.590)_ = 0.6249, *p* = 0.583; 3 s, *F*_(4.487,53.8)_ = 2.150, *p* = 0.08; 4 s, *F*_(1.080,2.159)_ = 4.808, *p* = 0.1516) as well as in reaction times (1.5 s, *F*_(2.667,13.33)_ = 0.766, *p* = 0.5189; 2 s, *F*_(1.966,7.862)_ = 0.8525, *p* = 0.4604; 3 s, *F*_(3.809,45.71)_ = 0.5034, *p* = 0.7247; 4 s, *F*_(1.398,2.796)_ = 0.8794, *p* = 0.4644) across different object pairs.

By changing the length of the delay period, we can manipulate the level of difficulty in each task. In the present study, we used four different delay lengths (1.5, 2, 3, and 4 s) in Monkey A and analyzed their impact on dual-task interference (Fig. 4). Regarding the SWM task, we observed a significant interference effect on the correct performance ratios across all delay lengths in the PA-first condition (−44% at 1.5 s, −42% at 2 s, −54% at 3 s, and −34% at 4 s; all *p* < 0.0001) as well as the SIM-PA-first conditions (−49% at 1.5 s, −43% at 2 s, −53% at 3 s, and −35% at 4 s; all *p* < 0.0001). However, in the SWM-first and SIM-SWM-first conditions, we only observed a significant interference effect at the 3 s delay lengths in the SWM-first condition (−17%, *t* = 3.072, *p* < 0.05). On the other hand, for the PA task, we found a notable interference effect on correct performance ratios at the 1.5 s (−15%), 2 s (−17%), and 3 s (−33%) delay lengths in the SWM-first condition (all *p* < 0.0001). Any significant differences in correct performance ratios were not observed across different spatial conditions in the SWM task and across different object pairs in the PA task (Fig. 5). Thus, the reduction in the correct performance ratio of the SWM task was more evident in the PA-first and SIM-PA-first conditions compared with the PA task in the SWM-first and SIM-SWM-first conditions, showing a greater impact of interference effect on the SWM task than on the PA task. The effect of task difficulty was more obvious in the PA task than the SWM task. The reason why the effect of task difficulty was not obvious in the SWM task in the PA-first and the SIM-PA-first conditions might be that the performance of the SWM task was the bottom level even in the 1.5 s delay condition since the interference effect by the PA task was strong. On the other hand, since the PA task was more difficult than the SWM task, the impact of the interference effect on the PA task from the SWM task increased as the difficulty of the PA task increased.

10.1523/ENEURO.0542-24.2025.f4-1 Figure 4-1 Effect of task difficulty on reaction times in the SWM task and the PA task under different task conditions in monkey A. Task difficulty was controlled by changing the delay length between 1 s and 4 s. Reaction time data for each delay condition were obtained in separate blocks of trials. Download Figure 4-1, TIF file.

We also examined reaction times of saccadic eye movements across the four different delay lengths for Monkey A (Extended Data Fig. 4-1). We observed an increase in reaction times for the SWM task in the SWM-first (+68.1 ms at 2 s and +58.1 ms at 3 s; all *p* < 0.01), the SIM- SWM-first conditions (+78.7 ms at 1.5 s, +84.9 ms at 2 s and +58.5 ms at 3 s; all *p* < 0.05), and the SIM-PA-first conditions at 3 s (+95.5 ms; *p* < 0.0001) but decrease in the SIM-PA-first conditions for 1.5 s (−80.4 ms at 1.5 s; *p* < 0.01) and 4 s (−84.4 ms; *p* < 0.05). The increase in reaction times for the PA task was evident in the all for dual-task conditions for the 1.5 s (+52.0 ms vs +73.0 ms vs +91.7 ms vs +113.3 ms; *F*_(4,21)_ = 14.04, *p* < 0.0001) and 2 s (+34.0 ms vs +42.2 ms vs +47.1 ms vs +59.3 ms; *F*_(4,22)_ = 9.313, *p* < 0.0001) delay lengths, and there was significant differences between the PA-first conditions (+51.4 ms), the SIM-SWM-first conditions (+53.4 ms), and the SIM-PA-first conditions (+80.5 ms; *F*_(4,60)_ = 26.90, *p* < 0.0001) in the 3 s delay lengths. No significant differences were noted in reaction times across different spatial conditions in the SWM task and across different object pairs in the PA task (Fig. 5). These results indicate that the interference effect increases as task difficulty increases and that reaction times of the SWM task were more affected than those of the PA task as task difficulty increased.

### Errors observed in dual-task conditions

**Figure 6.**
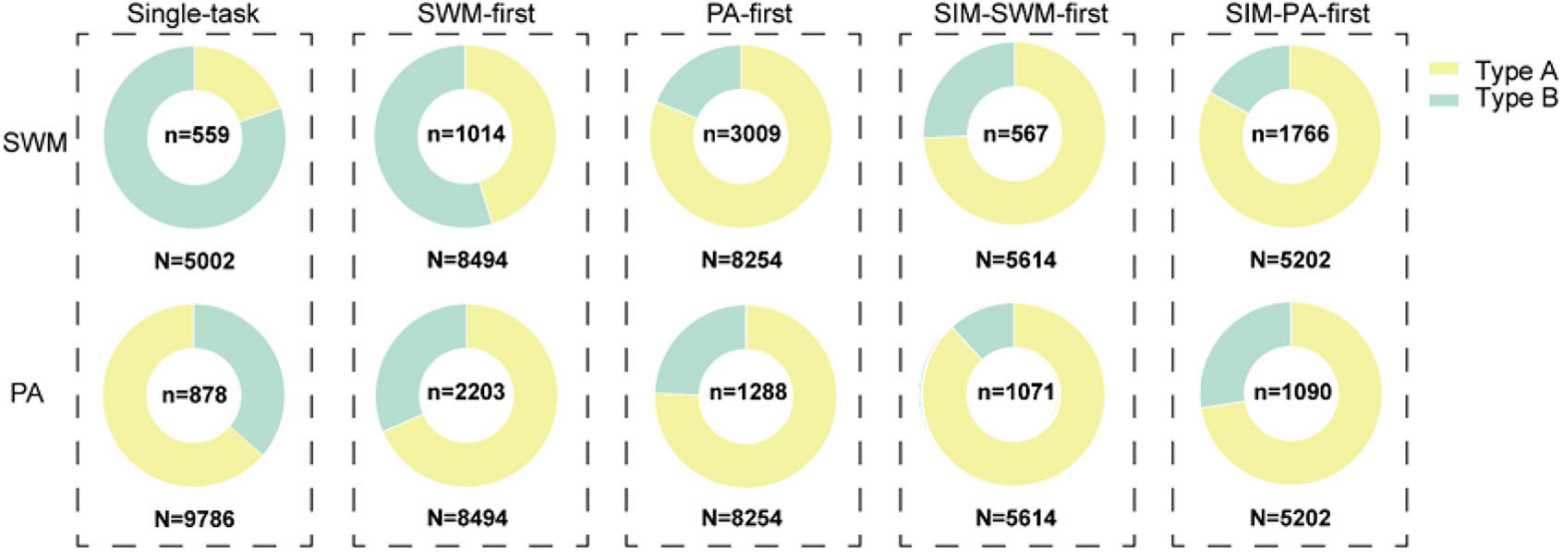
Error types observed in the SWM task and the PA task among different task conditions in Monkey A. Two types of errors (type A and B) were detected. Type A is an error that the subject failed to make a saccade to the correct target position within 0.5 s in the SWM task or failed to catch the correct target object within 0.8 s in the PA task. Type B is an error that the subject failed to maintain gazing on the spatial target position for 0.5 s in the SWM task or the correct target object for 0.5 s in the PA task. Total number of examined trials is shown under each circular graph (e.g., *N* = 4,433) and total number of errors observed in each task under each condition is shown at the center of each circular graph (e.g., *n* = 588). Different colors in each circular graph show proportions of type A errors (yellow) and type B errors (blue).

Error ratios and types of errors were compared between single-task and dual-task conditions. Error ratios of the SWM task were 11% in single-task condition, 12% in SWM-first, 36% in PA-first, 10% in SIM-SWM-first, and 40% in SIM-PA-first (Fig. 6). On the other hand, error ratios of the PA task were 23% in single-task condition, 30% in SWM-first, 16% in PA-first, 19% in SIM-SWM-first, and 21% in SIM-PA-first (Fig. 6). These results indicate that behavioral performances of the PA task were not affected by whether the SWM task were preceded or followed (*p* = 0.590, *t* test). However, the behavioral performances of the SWM task were strongly affected when the PA task was preceded (*p* = 0.070, *t* test). These results indicate an importance of the task order in dual-task interference. These results also suggest an importance of task features in dual-task interference.

Types of errors were also compared between single-task and dual-task conditions (Fig. 6). During the response period, monkeys were required to make a saccade to the spatial target position within 0.5 s (SWM task) or to the target picture within 0.8 s (PA task) and then to keep looking at the target position or the target picture for 0.5 s (hold period). Two types of errors (types A and B) were observed. Type A error is the one that monkeys failed to catch the correct spatial position or the target picture within the max response period. Type B error is the one that monkeys could not keep looking at the target position or the target picture during the hold time. For the PA task, type A error occupied larger proportion in the single-task condition (64%) and all dual-task conditions (SWM-first, 68%; PA-first, 76%; SIM-SWM-first, 88%; SIM-PA-first, 72%) compared with type B error (*p* = 0.04, *t* test). On the contrary, for the SWM task, type B error occupied larger proportion in single-task (80%) compared with type A error (20%), while type A error occupied a large proportion in PA-first (81%) and SIM-PA-first conditions (83%) compared with type B error (*p* = 0.020, *t* test). In the SWM-first condition, proportion of type A error (45%) was similar as type B error (55%). Since monkeys needed to maintain spatial information of the visual target and catch the target by an eye movement in the SWM task, these results suggest that object memory for the PA task strongly interferes spatial memory for the SWM task while object memory for the PA task is not much affected by spatial memory for the SWM task.

### SWM task and DMS task in dual-task condition

**Figure 7.**
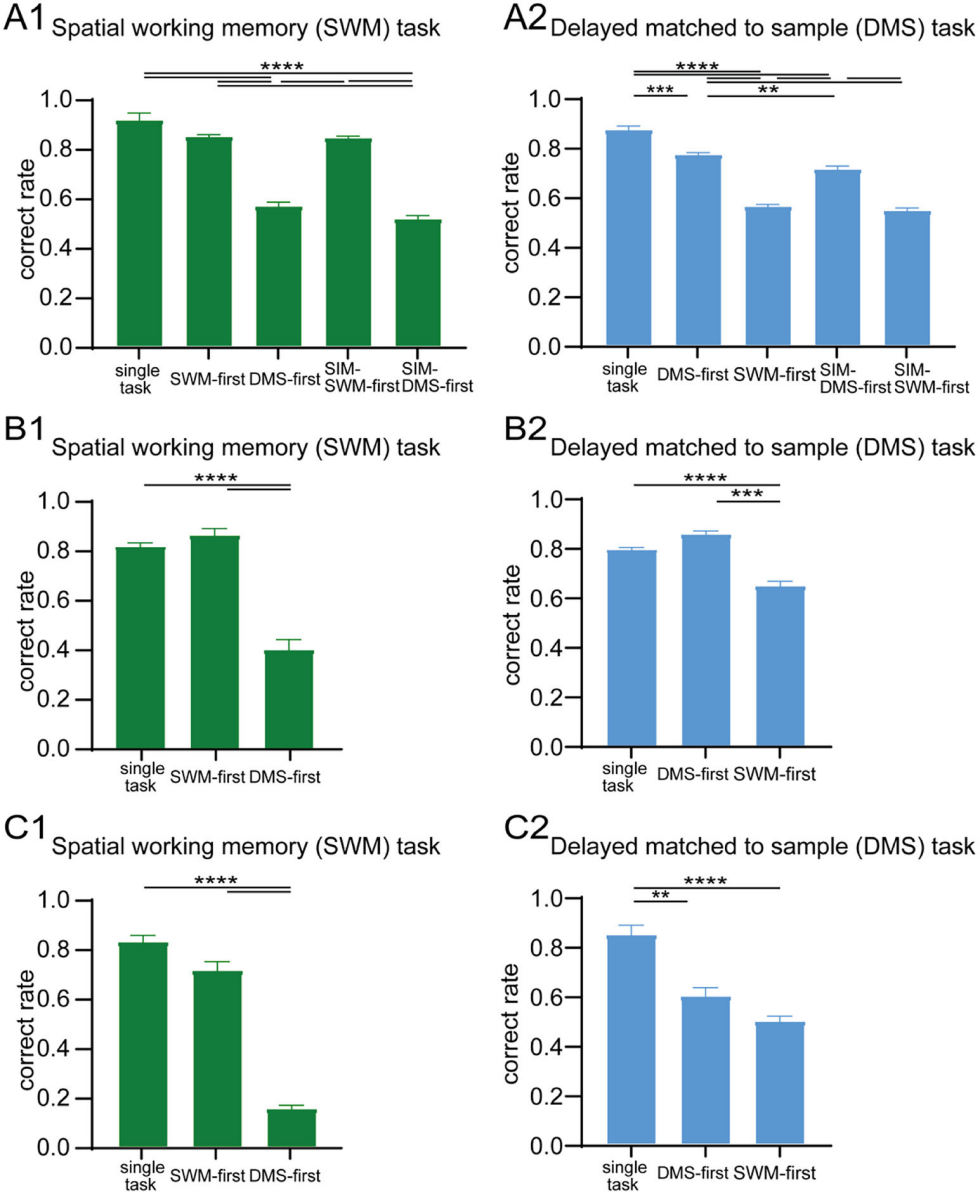
Behavioral performances in single-task and dual-task conditions for three monkeys when the SWM task and the delayed matching-to-sample (DMS) task were used as component tasks. ***A1–C1***, Comparison of correct performance ratios of the SWM task among different task conditions for Monkeys A, B, and C. ***A2–C2***, Comparison of correct performance ratios of the DMS task among different task conditions for Monkeys A, B, and C. The delay length was 3 s for all task conditions. *p < 0.05, **p < 0.01, ***p < 0.001, ****p < 0.0001.

The performance of the PA task requires to retrieve its paired associate from long-term memory and to maintain the retrieved object in working memory. In contrast, the performance of the SWM task relies solely on the retention of the spatial position of the visual cue in working memory. To examine whether the strength of dual-task interference effect is different between a task required object working memory and a task required spatial working memory, the PA task was replaced with a delayed matching-to-sample (DMS) task using eight colored object stimuli (Extended Data Fig. 1-1), and dual-task interference effects were examined using the SWM task and the DMS task as component tasks in dual-task conditions. In single-task condition, average ratios of correct performances for Monkeys A, B, and C were 91%, 81%, and 82% in the SWM task and 87%, 79%, and 84% in the DMS task, respectively, suggesting that the difficulty of the DMS task is almost equal with the SWM task for these monkeys (Monkey A: *t* = 1.249, *p* = 0.2337; Monkey B: *t* = 1.934, *p* = 0.0722; Monkey C: *t* = 0.3489, *p* = 0.7324; Fig. 7).

In dual-task conditions, for Monkey A, the correct performance ratio of the SWM task was significantly decreased in the DMS-first (56%) and the SIM-DMS-first (51%) conditions compared with single-task condition (91%; *F*_(2,44)_ = 5.761, *p* < 0.001; Fig. 7*A1*). Similarly, the correct performance ratio of the DMS task was significantly decreased in the SWM-first (56%) and the SIM-SWM-first (57%) condition compared with single-task condition (87%; *F*_(2,45)_ = 131.3, *p* < 0.0001; Fig. 7*A2*). Similar results were also observed in Monkeys B and C. Correct performance ratio of the SWM task was significantly decreased in the DMS-first condition (39% in Monkey B and 17% in Monkey C; Monkey B: *t* = 8.929, *p* < 0.0001; Monkey C: *t* = 17.59, *p* < 0.0001; Fig. 7*B1*,*C1*). Correct performance ratio of the DMS task was also decreased in the SWM-first condition (64% in Monkey B and 49% in Monkey C; Monkey B: *t* = 5.658, *p* < 0.0001; Monkey C: *t* = 6.58, *p* < 0.0001; Fig. 7*B2*,*C2*). In dual-task experiments involving SWM and PA tasks, the performance of the SWM task was more affected when it was introduced after the PA task (Fig. 2). Similarly, when SWM and DMS tasks were employed, the task performed second was impaired. The performance of the SWM task was more strongly affected when the DMS task was introduced first, especially in Monkeys B and C (Fig. 7). This indicates that, although the task order plays a role in determining the dominant task, the difference of information necessary to perform the task (space or object) plays more significant role to determine the dominant task in dual-task conditions.

## Discussion

The present study used monkeys and investigated behavioral factors determining the dominant task in dual-task conditions, with three different tasks requiring distinct types of information processing (SWM, spatial working memory; DMS, object working memory; PA, object working memory and long-term memory of object pairs). The task order (e.g., SWM-first, PA-first), fixed-order or random-order, and task difficulty were manipulated. As a result, the correct performance ratio of the first task was better than that of the second task. This result was observed regardless of which tasks were used as component tasks in dual-task conditions, indicating the task order as an important task factor to determine the dominant task. However, when the SWM and the PA tasks were introduced secondly, the reduction of the correct performance ratio was greater in the SWM task than the PA task, indicating a stronger dual-task interference effect on the SWM task. The overall performance of the SWM task was better than the PA task in single-task conditions, suggesting that the SWM task is easier than the PA task. However, negative effect caused by dual-task interference was stronger in the SWM task than the PA task. This tendency was more obvious in random-order conditions than fixed-order conditions. Similarly, when the SWM and DMS tasks were introduced secondly, the strength of dual-task interference effect was stronger in the SWM task than the DMS task, in spite that correct performance ratios were similar between these tasks in single-task conditions. Although the task order is one determinant, present findings emphasize the importance of the specific behavioral tasks employed as component tasks, whether they share common cognitive processes or not, their complexity and difficulty, and switching and planning tasks (fixed-order vs random-order) as more critical task factors to determine the dominant task in dual-task conditions. The present results indicate that the cause of dual-task interference is closely associated with the neural mechanisms of executive control (Shallice and Burgess, 1991; Meyer and Kieras, 1997; Baddeley and Della Sala, 1998; Smith and Jonides, 1999), because managing working memory resources and coordinating multiple cognitive processes are essential mechanisms for both dual-task interference and executive control.

### Effect of task order

The current study used SWM, PA, and DMS tasks as component tasks to investigate the effect of dual-task interference by altering the order of the two tasks in either fixed-order or random-order conditions. The results revealed a task order effect in the dual-task condition, where the performance of the first task consistently outperformed the second task in fixed-order conditions, regardless of the specific component tasks used. This task order effect has been previously observed in dual-task experiments using the psychological refractory period (PRP) paradigms (Pashler, 1994; Strobach and Torsten, 2017). In this paradigm, two stimuli (S1 and S2) are presented rapidly with a variable SOA and the subject needs to perform corresponding responses (R1 and R2) in the same order as the stimulus presentation. It has been shown that, when the SOA increases, reaction times of R2 become short, while reaction times of R1 remains constant (Pashler, 1994; Strobach and Torsten, 2017). This phenomenon has been attributed to the bottleneck model, which suggests that if two component tasks share a single common neural process for information processing, only one task can access that process at a given time in the dual-task condition. Consequently, when the neural process is actively engaged in information processing for the first task, processing for the second task is inevitably suspended until the neural process becomes available, resulting in a bottleneck. The order of arrival at this bottleneck determines the processing order, following the principle of first-come-first-serve (De Jong, 1995; Strobach and Torsten, 2017).

Studies have shown that dual-task interference is more pronounced when tasks share common features or common cognitive processes (Cocchini et al., 2002; Fougnie and Marois, 2006; Baddeley, 2012). For example, previous studies have demonstrated stronger interference effects between memory sets within the same information domain (e.g., visual) compared with sets from different information domains (e.g., visual vs verbal; Cowan and Morey, 2007). In the present experiment, stronger interference effects were observed in the SWM task under PA-first and DMS-first conditions, as well as in the PA and DMS tasks under the SWM-first conditions, possibly due to the shared reliance on working memory resources for visual information processing in all tasks involved. Although the task order effect observed in PRP paradigms has been primarily explained by the idea that the common response-selection process cannot be simultaneously utilized for two tasks (Pashler, 1994), Jiang (2004) demonstrated that the two component tasks not only competed at the response-selection process but also at the perceptual and attention processes, suggesting the presence of a bottleneck at both perception and response processes. The SWM, PA, and DMS tasks all share two common neural processes, one for maintaining visual information during the delay period (visual working memory) and another for controlling eye movements during the response period. Therefore, the task order becomes a critical factor in determining which task becomes the winner when two component tasks share a common neural process for information processing. Especially in the fixed-order condition, the SWM-cue and PA-cue were presented sequentially for 250 ms without an interval, and the SWM-response and PA-response were performed in the same order as the presentation of these cues, similar to the PRP paradigm. Since both the SWM and PA tasks share common neural processes for visual working memory and eye movement control, the second task can only access these neural processes until the neural processes for the first task are completed in the dual-task condition. Consequently, the task order effect observed in the fixed-order conditions could be elucidated by the bottleneck model.

Neurophysiological experiments with monkeys have provided compelling evidence for the task-order effect of dual-task interference. Watanabe and Funahashi (2014) used a spatial working memory task and a spatial attention task as component tasks for dual-task experiments. They discovered that the interference effect was much stronger on the secondly introduced working memory task compared with the first introduced attention task. Moreover, they observed that delay-period activity, which was evident in the working memory task under single-task conditions, significantly diminished in dual-task conditions until the attention task was completed. This indicated that the engagement of the spatial working memory process in the first attention task hindered the second working memory task from utilizing the process until it became available. When the first attention task was completed, “awakening” of delay-period activity was observed (Watanabe and Funahashi, 2014; Funahashi, 2022b; Watanabe et al., 2023), leading to an activation pattern that aligned with the bottleneck model's predictions. Although the researchers did not directly investigate neural activity under reversed order conditions, their findings strongly support the notion that the performance of the first task can dampen task-related activities for the second task, underlining the pivotal role of task order in determining the dominant task in dual-task conditions.

However, in random-order (SIM) conditions, both a spatial cue and an object cue were presented simultaneously, and the subject could not predict which response was needed first. Behavioral results showed that the performances of the PA task and the DMS task were less affected in the SWM-first and the SIM-SWM-first conditions. However, the performance of the SWM task was strongly affected in SIM-PA-first and SIM-DMS-first conditions. Negative effect caused by dual-task interference was stronger in the SWM task than the PA and DMS task. Kubler et al. (2022) compared behavioral performances between fixed- and random-order conditions and showed that behavioral performances deteriorated in random-order conditions, suggesting that the task order effect is not solely attributed to the bottleneck mechanism but also to working memory mechanisms for maintaining task-order information. Schumacher et al. (2001) and Schumacher and Schwarb (2009) indicated an importance of central cognitive bottleneck in sequence learning under dual-task conditions, which affects task priorities and task scheduling. In the SIM conditions, information regarding which of two tasks performed first was provided only at the beginning of the response period. Therefore, the subjects needed to promptly select the task and retrieve necessary information for the first response. Although the task order is an important task factor, the results obtained in the random-order conditions suggest that task factors other than the task order, such as executive processes for promptly switching and coordinating the performance of two concurrent tasks adaptively (Meyer and Kieras, 1997; Hazeltine, 2024), play more critical roles to decide which task becomes the winner in dual-task conditions.

### Capacity effects

It is widely recognized that the capacity of resources for a variety of cognitive information processing is limited (Kahneman, 1973; Wickens, 2002). The capacity of working memory resources has been extensively studied and found to be limited (Engle et al., 1999; Cowan, 2001; Marois and Ivanoff, 2005; Baddeley, 2012). Tasks such as SWM, PA, or DMS all require a certain amount of working memory resources to perform effectively. Consequently, the amount of working memory resources needed for each task is a crucial factor in dual-task conditions, as exceeding the total available working memory resources can lead to dual-task interference when two tasks compete for the same finite resources simultaneously. Generally, tasks involving more complex and multiple neural processes are expected to demand a larger amount of cognitive resources compared with tasks that are simpler and involve a single process. Moreover, differences in task difficulty are anticipated to impact the necessary amount of cognitive resources, with more difficult tasks requiring greater cognitive resource allocation than easier tasks. In the present study, the tasks varied in difficulty and complexity and the number of neural processes involved. For instance, the PA task requires both long-term memory of paired objects and object working memory, while the SWM and DMS tasks require spatial and object working memory, respectively. Thus, the difficulty and the complexity would be important task factors affecting which task becomes the winner, because these task factors determine the amount of working memory resources necessary to perform the tasks.

In dual-task conditions, simultaneous retention of the information necessary for the accurate completion of both tasks is demanding. Interestingly, regardless of the order in which the tasks are performed, their performance is influenced by the overlap of retaining information for one task while simultaneously retrieving information for the other. Fougnie and Marois (2009) conducted a study using a dual-task paradigm, which involved the concurrent use of working memory for two different visual arrays. Their findings indicated that retaining information for one visual array did not interfere with the retrieval of information from another, unless the combined information exceeded the capacity of working memory. Anderson et al. (2010) showed that, when inefficient visual search (rotated T among Ls) was performed concurrently with either a spatial or a nonspatial working memory task, search efficiency declined in a comparable degree for both tasks, suggesting that working memory tasks are highly demanding of resources to maintain, update, retrieve, and select information. Interestingly, Postle et al. (2005) suggested that the retention of visual object and spatial information differs in terms of their representation in working memory. Repovs and Baddeley (2006) further divided visuospatial working memory into spatial and object subsystems, proposing that each subsystem has distinct storage, maintenance, and manipulation processes. In object working memory, different features of an object are maintained independently, with the number of features that can be retained simultaneously depending on the maximum number of distinct features an object possesses, such as colors, shapes, and line orientations. It is well established that the maximum storage capacity of working memory is around four items (Engle et al., 1999; Cowan, 2001), a capacity that is similar in both humans and monkeys (Buschman et al., 2011). Since the number of items retained in working memory for the SWM task is smaller than that for the PA and DMS tasks, a larger allocation of working memory resources is needed for the PA and DMS tasks compared with the SWM task. Although task-order effect was present, the performance of the SWM task was more strongly affected in the SIM-PA-first and SIM-DMS-first conditions, while the performance of the PA and DMS tasks remained largely unaffected in the SIM-SWM-first condition, indicating that the demand of working memory resources is larger in the PA and DMS tasks than the SWM task. This suggests that the amount of cognitive resources required for a task is an important determinant for the dominant task in dual-task conditions, with the task that requires a larger amount of resources emerging as the dominant task.

Although the amount of cognitive resources required for retaining information during the delay period is an important determinant, cognitive resources required for encoding or retrieving information may also affect the dominant task in dual-task conditions. In the SIM conditions, both a spatial cue and an object cue were presented simultaneously during the 250 ms cue period, and correct performance ratios at the first response in SIM conditions (the SWM task in the SIM-SWM-first condition and the PA task in the SIM-PA-first condition) were comparable with correct performance ratios of these tasks in single-task conditions, suggesting that the amount of cognitive resources required for encoding spatial or object information is not an important determinant. However, during the response period, the performances of the PA task and the DMS task were less affected in the SIM-SWM-first conditions as well as the SIM-PA-first and the SIM-DMS-first conditions compared with their single-task conditions. However, the performance of the SWM task was not much affected in the SIM-SWM-first conditions but was strongly affected in SIM-PA-first and SIM-DMS-first conditions, suggesting that the retrieval of object information for PA and DMS tasks interferes retention and retrieval of spatial information for the SWM task. Thus, how much working memory resources are allocated to each component tasks for retaining as well as retrieving necessary information is an important determinant of which task becomes a winner in dual-task conditions.

### Difference of information processing between component tasks

In the preset study, three distinct cognitive tasks were employed, each demanding unique sets of information processing and engaging different neural mechanisms. The SWM task required retention of spatial information, the DMS task required retention of object information, and the PA task required working memory of object information combined with long-term memory of object pairs. Previous studies on dual working memory tasks have demonstrated a stronger interference effect between memory sets within the same information domain (e.g., visual) compared with sets across different information domains (e.g., visual vs verbal; Cowan and Morey, 2007). Studies have provided evidence for the existence of separate storage systems for visual objects and spatial information within working memory (Klauer and Zhao, 2004; Repovs and Baddeley, 2006). For instance, experiments conducted by Klauer and Zhao (2004) revealed that object-based working memory tasks were more susceptible to object interference, whereas spatial working memory tasks were more disrupted by spatial interference, indicating distinct visual and spatial storage mechanisms through double dissociation. Object working memory is closely related to perception and visual imagery, while spatial working memory is more associated with attention and oculomotor control. Postle et al. (2006) suggested that the disruptive effect on spatial working memory is primarily linked to eye movement control rather than the movements themselves and is specific to spatial working memory, not extending to visual working memory. These findings suggest that spatial and object working memory tasks rely on separate and independent neural processes. In addition, neural mechanisms for working memory are separate and distinct from those supporting long-term memory functions. Consequently, differences in interference effects observed between the spatial task (SWM) and the object tasks (PA and DMS), as well as between the working memory task (SWM) and the task involving long-term memory (PA) in this dual-task experiment, could be attributed to variations in the neural mechanisms underpinning each task.

Given the distinct information processing mechanisms of each sensory system, it is possible to observe varying levels of interference in dual-task conditions involving different sensory systems. Kasper et al. (2014) employed multisensory continuous dual tasks with participants exposed to rapid visual and auditory stimulus sequences concurrently, under three distinct conditions: focusing solely on the visual stimuli, solely on the auditory stimuli, or on both visual and auditory stimuli simultaneously. The analysis of behavioral performance metrics such as response time and perceptual sensitivity revealed that dual-task interference effect was only evident in the auditory task. Furthermore, the study demonstrated that while auditory sensory and cognitive processes, as assessed through event-related potentials, were affected by dual-task demands, visual processes remained unaffected. These findings indicate a tendency for the visual task to become the winner in dual-task conditions involving visual and auditory stimuli, indicating that the sensory modalities used can influence task prioritization. However, when the auditory task requires more cognitive resources than the visual task (e.g., tasks involving word generation or memorization of auditory passages), the auditory task emerges as the dominant task in dual-task conditions (Gherri and Eimer, 2011). This indicates the significance of cognitive load in determining the dominant task in dual-task conditions, emphasizing the critical role of memory management as well as coordination of multiple cognitive processes.

## Data Availability

This study did not generate new unique reagents. All data are included in the article and supporting information. Any additional information for this paper is available from the leading contact upon request.

## Synthesis

Reviewing Editor: Mark Laubach, American University Decisions are customarily a result of the Reviewing Editor and the peer reviewers coming together and discussing their recommendations until a consensus is reached. When revisions are invited, a fact-based synthesis statement explaining their decision and outlining what is needed to prepare a revision will be listed below. The following reviewer(s) agreed to reveal their identity: Wenhao Dang.

Thank you for sending your work to eNeuro!

Your paper was reviewed by two experts. Their reviews are given below. Please revise to address all points that were raised. Please take special effort to address the comments of the second reviewer, who felt that your literature review was not sufficient.

Reviewer 1

Nonhuman primates were trained to perform both single and dual working memory tasks that require different cognitive processes. Behavioral performance was compared between the single-task condition and its counterpart when tasks were combined in a dual-task setting. The authors successfully identified multiple determinants that influence which task is prioritized in this dual-task paradigm. This study is particularly interesting, as complex working memory paradigms are rarely used in animal research, and the findings will likely appeal to a broad audience. However, the manuscript would benefit from further discussion on several points.

Major Comments:

1. In a dual-task working memory paradigm, two primary cognitive processes can influence performance: encoding and retrieval. Poor performance may result from either lower-quality encoding due to limited cognitive resources or reduced resilience to interference caused by the readout of information from the competing task. The paper appears to address working memory processes in general, without distinguishing between these two factors. The current task design could potentially provide insights into this issue, particularly by comparing the fixed-order and random-order conditions. The random-order task, in particular, may isolate the factor of resilience to interference from the competing task.

2. While the current study provides valuable data from nonhuman primates on complex working memory tasks, the inherent limitation of small sample sizes in nonhuman primate research can lead to variability in results. For instance, in Figure 3, the reaction time trends differ for Animal A and Animal B, and in Figure 4, the impact of the second task on SWM performance does not seem consistent across different delay intervals. Additionally, nonhuman primate behavior is often influenced by training history or individual strategies employed by the subjects. For example, in a simultaneous working memory task, different animals may prioritize information based on personal preferences or past experiences, making it difficult to ascertain the strategies employed by the animals. It would be valuable to include supplementary human psychophysical experiments, and the authors should address the potential impact of individual strategies and training history in the discussion.

Minor Comments:

1. The statistics for the proportion in Figure 6 are not provided. The inclusion of this information would help clarify the results.

2. In the results section, the authors claim that interference has a greater impact on the SWM task compared to the PA task. How is this interference quantified? Although the proportion drop for SWM (from ~95% to ~40%) is larger than for PA (from ~84% to ~50%), it is important to note that the baseline performance for the two tasks is different, with the PA task having a lower ceiling. This difference should be addressed and clarified.

Reviewer 2

The manuscript uses non-human primates to investigate dual-task processing. This is an interesting extension of work typically done with humans. The manuscript cites some important literature on human dual-task processing, but ignores other relevant literature (Anderson; Hazeltine; Meyer &Kieras; Schumacher). Meyer &Kieras' work on the strategic causes of dual-task interference seems especially relevant to the current topic.
