# Supplementary figures and images for: What Task Feature Determines the Dominant Task in Dual-Task Conditions?
Source: eNeuro. 2025 Mar 19;12(3):ENEURO.0542-24.2025. doi: 10.1523/ENEURO.0542-24.2025 (PMC11949648; doi:10.1523/ENEURO.0542-24.2025)

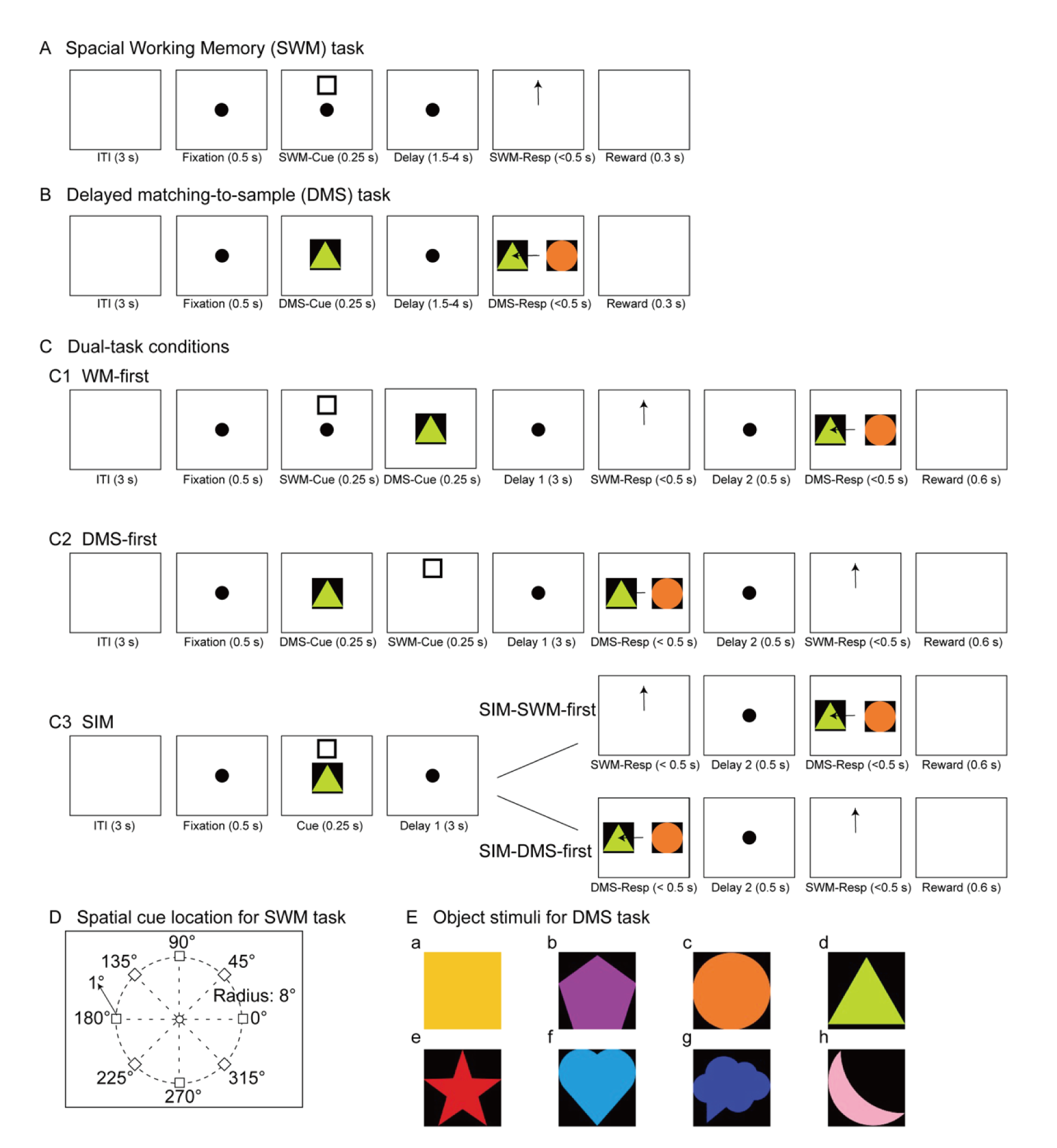

Supplement: Figure 1-1 — Illustration of the experimental paradigms used the SWM task and the DMS task as component tasks. (A) Spatial working memory (SWM) task in single-task condition. This task requires to maintain a spatial cue position during the delay period. (B) Object delayed matching-to-sample (DMS) task in single-task condition. This task requires to maintain the object cue after the delay period. (C) Dual-task conditions using SWM and DMS tasks. Dual-task conditions include two fixed-order conditions (SWM-first and DMS-first) and two random-order (SIM) conditions (SIM-SWM-first and SIM-DMS-first). In fixed-order conditions, SWM-cue and DMS-cue were presented sequentially in fixed order either in SWM-cue first or DMS-cue first, and the subject performed eye movement responses with the same order as the cue presentation. In random-order conditions, both SWM-cue and DMS-cue were presented simultaneously, and subjects performed either SWM task or DMS task first depending on whether no visual stimulus was presented (SIM-SWM-first) or two object stimuli were presented (SIM-DMS-first) on the monitor after the delay period. (D) Positions of the spatial cue for the SWM task. (E) Eight objects for the DMS task. Arrows in figures indicate directions of correct eye movements. Download Figure 1-1, TIF file. [file eneuro-12-ENEURO.0542-24.2025-s002.tif]

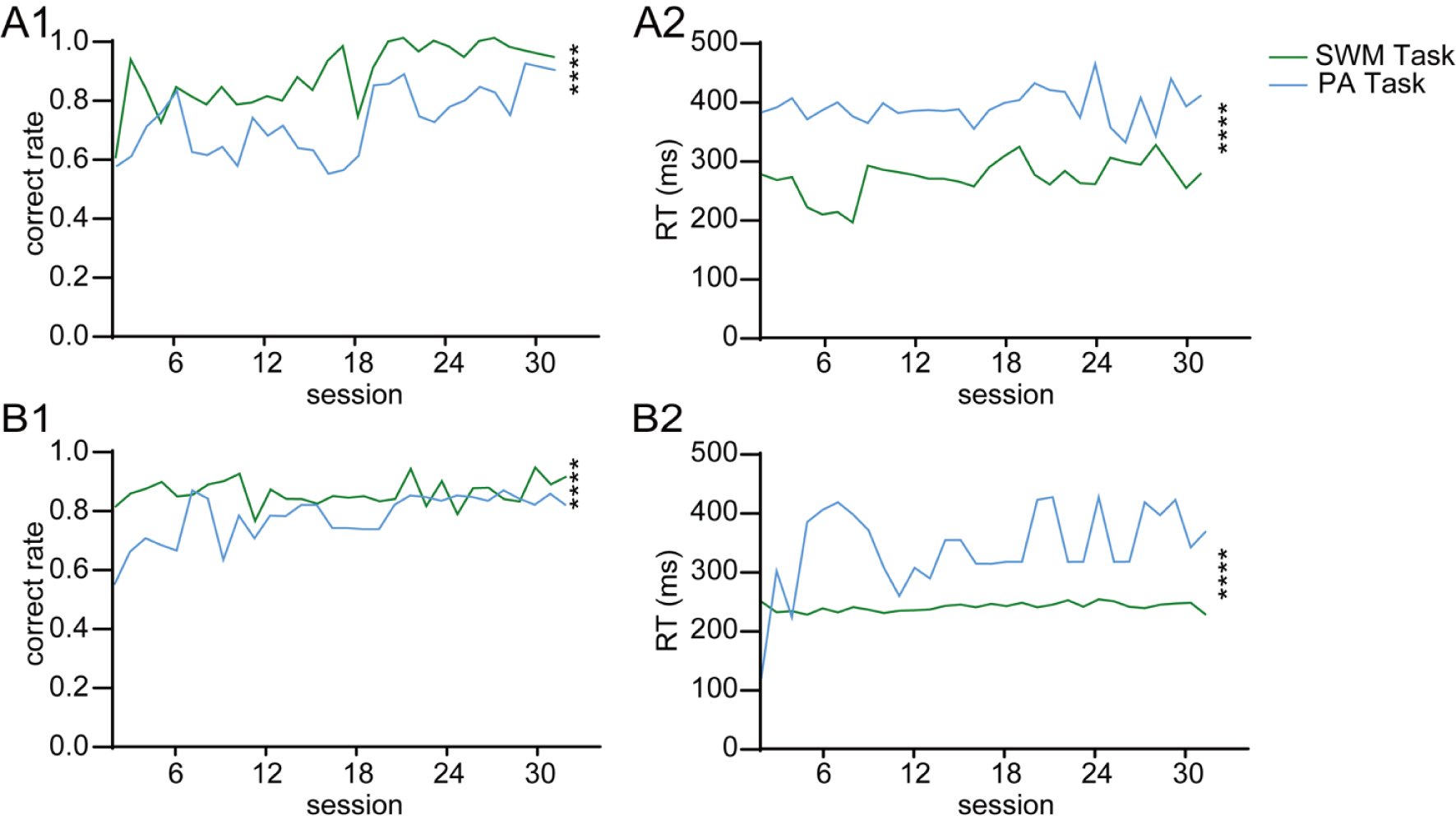

Supplement: Figure 2-1 — Temporal changes of behavioral Performances of both SWM and PA tasks along different experimental sessions. (A1 and A2) Correct performance ratios (A1) and reaction times (A2) in the SWM task (green) and the PA task (blue) for monkey A. (B1 and B2) Correct performance ratios (B1) and reaction times (B2) in the SWM task (green) and the PA task (blue) for Monkey B. **** p < 0.0001. Download Figure 2-1, TIF file. [file eneuro-12-ENEURO.0542-24.2025-s003.tif]

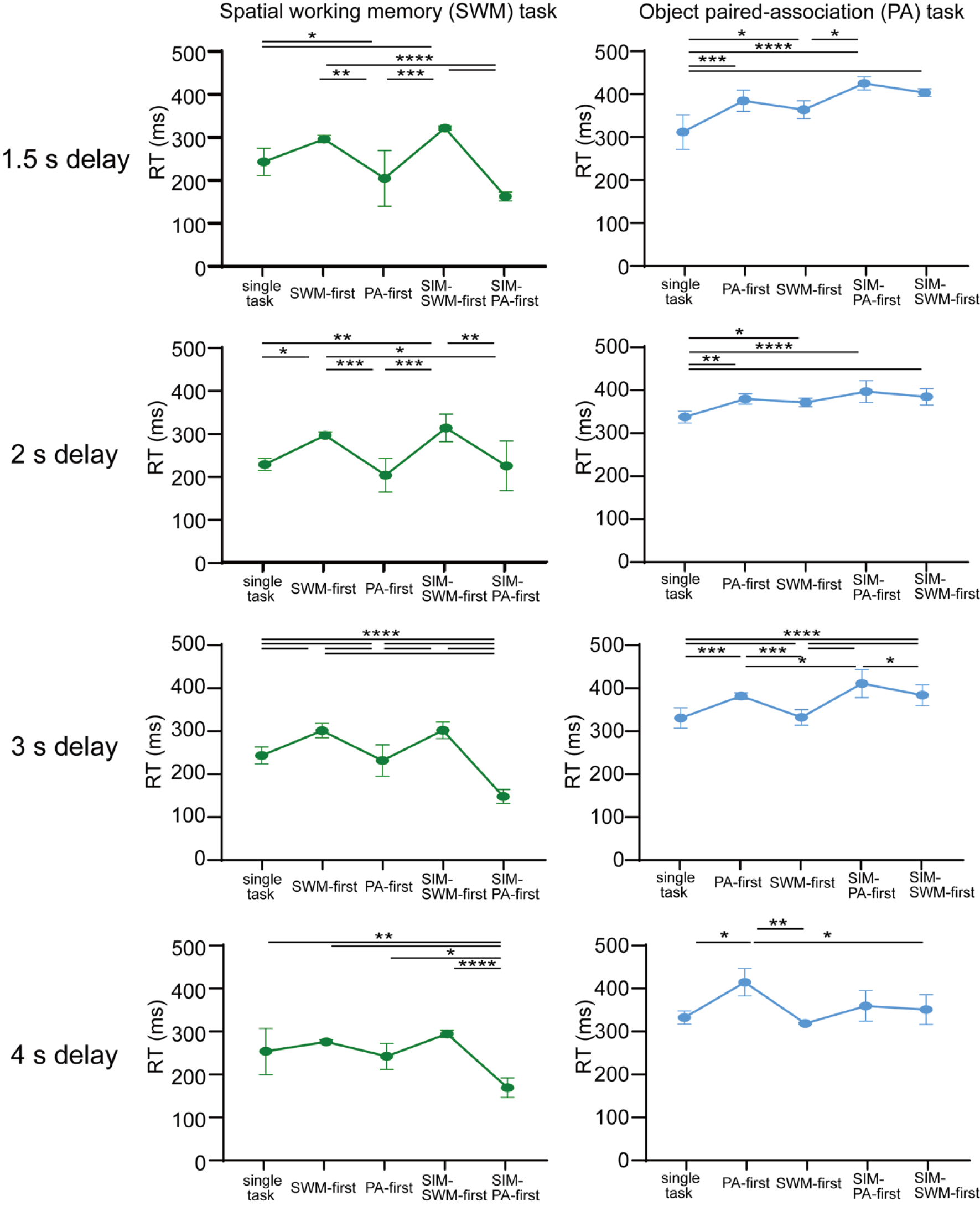

Supplement: Figure 4-1 — Effect of task difficulty on reaction times in the SWM task and the PA task under different task conditions in monkey A. Task difficulty was controlled by changing the delay length between 1 s and 4 s. Reaction time data for each delay condition were obtained in separate blocks of trials. Download Figure 4-1, TIF file. [file eneuro-12-ENEURO.0542-24.2025-s004.tif]
